# An heuristic filtering tool to identify phenotype-associated genetic variants applied to human intellectual disability and canine coat colors

**DOI:** 10.1186/s12859-015-0822-7

**Published:** 2015-11-19

**Authors:** Bart J. G. Broeckx, Frank Coopman, Geert Verhoeven, Tim Bosmans, Ingrid Gielen, Walter Dingemanse, Jimmy H. Saunders, Dieter Deforce, Filip Van Nieuwerburgh

**Affiliations:** 1 Laboratory of Pharmaceutical Biotechnology, Faculty of Pharmaceutical Sciences, Ghent University, 9000 Ghent, Belgium; 2 Department of Applied Biosciences, Faculty of Bioscience Engineering, Ghent University, 9000 Ghent, Belgium; 3 Department of Medical Imaging and Small Animal Orthopaedics, Faculty of Veterinary Medicine, Ghent University, 9820 Merelbeke, Belgium; 4 Department of Medicine and Clinical Biology of Small Animals, Faculty of Veterinary Medicine, Ghent University, 9820 Merelbeke, Belgium

**Keywords:** Dominant, Heuristic, Recessive, Sequence analysis, Variant filtering

## Abstract

**Background:**

Identification of one or several disease causing variant(s) from the large collection of variants present in an individual is often achieved by the sequential use of heuristic filters. The recent development of whole exome sequencing enrichment designs for several non-model species created the need for a species-independent, fast and versatile analysis tool, capable of tackling a wide variety of standard and more complex inheritance models. With this aim, we developed “Mendelian”, an R-package that can be used for heuristic variant filtering.

**Results:**

The R-package Mendelian offers fast and convenient filters to analyze putative variants for both recessive and dominant models of inheritance, with variable degrees of penetrance and detectance. Analysis of trios is supported. Filtering against variant databases and annotation of variants is also included. This package is not species specific and supports parallel computation. We validated this package by reanalyzing data from a whole exome sequencing experiment on intellectual disability in humans. In a second example, we identified the mutations responsible for coat color in the dog. This is the first example of whole exome sequencing without prior mapping in the dog.

**Conclusion:**

We developed an R-package that enables the identification of disease-causing variants from the long list of variants called in sequencing experiments. The software and a detailed manual are available at https://github.com/BartBroeckx/Mendelian.

**Electronic supplementary material:**

The online version of this article (doi:10.1186/s12859-015-0822-7) contains supplementary material, which is available to authorized users.

## Background

The identification of genetic variation responsible for a phenotype, is one of the key aims in the field of genetics. This field has been revolutionized with the introduction of next generation sequencing technologies and is continuously evolving. Although several sequencing platforms exist, the analysis of sequencing data generated in disease-association studies is virtually identical: the platform-specific raw data is used for base-calling and subsequently for mapping and variant calling against a reference genome. These variants can subsequently be used to perform a disease-association analysis, where the typical aim is to identify one or several disease causing variant(s) from the large collection of variants present in an individual. This can be achieved by the sequential application of several heuristic filters [1].

As genetic diseases are heterogeneous, a wide range of filters is required. Compared to complex disorders, it is more straightforward to identify disease causing variants in Mendelian disorders. However, even in this subgroup of Mendelian disorders, a variety of factors might complicate the analysis: different inheritance models (dominant, recessive), de novo mutations, allelic or locus heterogeneity, reduced penetrance, phenocopies, etcetera [1].

Due to the recent development of whole exome sequencing (WES) enrichment designs for several non-model species, these species are likely to be sequenced more often [2–5]. To be of practical use, heuristic filtering software should thus be capable to deal with all the aforementioned situations for both model and non-model species. At this point however, most tools are specifically intended for human analyses and/or only allow the most basic filtering. This limits the broad application of sequencing based approaches as it requires access to bioinformaticians that have to write custom scripts for the analysis at hand. To avoid a constant reinvention of the wheel and to fulfill the need for a species-independent, fast and versatile analysis tool, capable of tackling a wide variety of inheritance models and complicating factors, we developed the R-package “Mendelian”. It allows the analysis of several types of variants, including single nucleotide polymorphisms, insertion-deletions and structural variants.

We demonstrate its validity in two practical examples. In the first example, we reanalyze the data of a human WES experiment that identified a *de novo* mutation responsible for intellectual disability [6]. The second example demonstrates the power of the combination of the exome-plus, a novel WES design in the dog, and Mendelian by revalidating the recessively inherited yellow and brown coat color phenotypes in the Labrador Retriever [5, 7–9]. This second analysis is also the first to use WES without prior mapping in the dog. The combination of WES and Mendelian is likely to aid future disease-association studies.

## Implementation

Flexibility of the applied software tool is an important aspect in disease-association studies as the species and phenotype studied might significantly alter the analysis process. For example, filtering steps might be omitted (e.g. when a variant database is not available for the studied species), the proposed inheritance model might be dominant or recessive and genetic heterogeneity might be present. An overview of the features of the tool is provided below. In addition, a detailed vignette is available together with the software package at the package website.

### Input

Mendelian allows for the use of the standard variant call format (VCF). In addition, specific .txt output from the commercial platform CLC Genomics Workbench is also supported. If necessary, variant files can be annotated using .bed or .gtf files. The variants can be assigned to a variety of units from standard databases, e.g. an exon or a gene. User-specific custom annotations can also be used.

### Filtering against variant databases

Often, the first step in filtering called variants consists of the removal of previously identified variants present in public databases such as dbSNP. This significantly reduces the number of putative variants. Depending on the disease studied, one can choose to use all the variants present in a database or to use only those variants that have a certain minor allele frequency (MAF). This step can be skipped if a dbSNP is not available for the species studied.

### Filtering sequencing variants

There are four variant filters to support both dominant and recessive modes of inheritance, filtering at the nucleotide level or at a user-defined level (often an exon or a gene). They can be applied on one or more affected individuals at once and allow for the inclusion of one or several unaffected control individuals.

The two (dominant and recessive) functions for filtering at the nucleotide level, consider individual variants at a single nucleotide position in the genome. Under a dominant mode of inheritance, no zygosity assumptions are made: every variant called in an affected individual is a putative disease causing variant. Every variant called in unaffected individuals can be used to filter the variants in affected individuals.

Under a recessive mode of inheritance, putative causal variants are assumed to be in a homozygous state. Only homozygous variants in unaffected individuals are used to filter variants in affected individuals.

The two functions for filtering at a user-specified level, consider the variants in a unit (e.g. an exon or a gene) together. This allows for allelic heterogeneity, which implies that different variants within one unit might be disease causing.

Under a recessive mode of inheritance, putative causal variants can both be homozygous and/or compound heterozygous. Compound heterozygosity means that an individual expresses a phenotype due to two different heterozygous alleles within a particular unit. Every unit with at least one homozygous variant or that is compound heterozygous, is retained. If several cases are available, the filter identifies shared units instead of shared nucleotides. Variants called in unaffected individuals are used to filter variants in cases in two consecutive steps. First, homozygous variants in controls are used for filtering. Next, all compound heterozygous variants within a unit are used for filtering.

Under a dominant mode of inheritance, no zygosity assumptions are made, resulting in every unit with at least one variant being retained in affected individuals. Every variant present in a control is used for filtering.

### Detectance and penetrance

All four filters allow for a reduced penetrance and reduced detectance. Penetrance is defined as the probability of seeing a certain phenotype, given the genotype. Detectance is defined as the probability of identifying a certain genotype, given the phenotype. A 100 % detectance and penetrance is often assumed. Under a reduced detectance, a causal variant can be identified, even under locus heterogeneity or when phenocopies are present. Under reduced penetrance, a causal variant can be present in an individual without the expression of the associated phenotype.

These theoretical definitions are translated into practice by Mendelian in two sequential steps. First, Mendelian calculates the possible detectance and penetrance levels using the following formulas:

$$ penetrance=\frac{n_c}{n_c+{c}_g} $$

$$ detectance = \frac{n_c}{n_c+{n}_d} $$

With for the phenotypically affected individuals:

*n*_*s*_ = {phenotypically “sick” animals (called “cases”)}; *n*_*c*_ = {phenotypically “sick” individuals with a shared (= “common”) genetic cause}; *n*_*d*_ = {phenotypically “sick” individuals with a different genetic cause or phenocopies} and *n*_*c*_ + *n*_*d*_ = *n*_*s.*_

and for the phenotypically unaffected individuals:

*c* = {phenotypically “healthy” animals (called “controls”)}, *c*_*g*_ = {phenotypically “healthy” animals with “sick” genotype}, *c*_*c*_ = {phenotypically “healthy” animals with “healthy” genotype} and *c*_*g*_ *+ c*_*c*_ *= c*. The relation between these abbreviations is depicted in detail in Table 1. By varying c_g_ (restrictions: 0 ≤ c_g_ ≤ c) for the penetrance and n_d_ (restrictions: 0 ≤ *n*_*d*_  <  n_s_) for the detectance over all the possible values, the different options are calculated and provided to the user to choose from.

**Table 1:** Relation between a genotype and a phenotype

		Phenotype	
		Affected	Healthy
Genotype	Affected	*n*_*c*_	*c*_*g*_
	Healthy	*n*_*d*_	*c*_*c*_
	Total	*n*_*s*_	*c*

*c*_*g*_ reflects the number of animals that have a reduced penetrance. *n*_*d*_ is the number of animals that have a different genetic cause and/or that are phenocopies. *n*_*c*_ are the animals that share a genetic cause and are phenotypically affected. *c*_*c*_ are the animals that are both genetically and phenotypically healthy. A priori, only *n*_*s*_ and *c* are known

After the user has chosen the appropriate levels of detectance and penetrance, c_g_ and n_c_ are calculated by rearranging both formulas:

$$ {c}_g=\frac{n_c}{penetrance}-{n}_c $$

And

$$ {n}_c= detectance\ .\ {n}_s $$

Practically, Mendelian assumes that under reduced penetrance a variant is allowed to be present in at most *c*_*g*_ phenotypical controls and that under reduced detectance the variant has to be present in at least *n*_*c*_ cases. The chosen penetrance and detectance levels are thus the lower limits, all variants with levels of penetrance and detectance at least as high will be returned by default. This can be adapted, if needed.

## Results and discussion

The output of the heuristic filters is a data frame that for each variant contains the chromosome, the exact location, the allele and the number of samples with that allele. To show the possibilities of Mendelian, we performed two separate analyses. All R commands used in this analysis are included [see Additional file 1]. All the data reanalyzed in this study was obtained from published studies that were approved by the institution’s ethical committees.

### Example 1: human intellectual disability

**Fig. 1:**
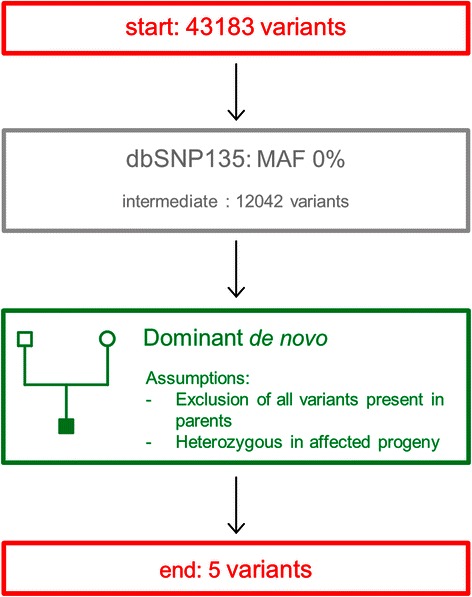
Consecutive filtering steps in the identification of putative causal variants for intellectual disability

As a starting point, we reanalyzed WES data from a study on intellectual disability [6]. A trio of one affected child and two healthy parents was sequenced and a *de novo* mutation was expected. Trio sequencing has the benefit that the vast majority of variants in the child will be present in at least one of the parents and with a *de novo* mutation, one can additionally assume that the variant has to be heterozygous in the affected child. This allows for an enormous reduction of variants, even though only three samples are sequenced. Two sequential filters were used in our analysis: after preprocessing, the VCF file containing the variants of the patient (patient #3 in the original study) was filtered against a human variant database. In agreement with the original study, the dbSNP135 was used with a MAF of 0 % (i.e. every variant in the database can be used for filtering). This already reduced the number of variants with 72.1 %. In the second filtering step the standard dominant filtering at the nucleotide level function was used, but with the “family” option specified. By specifying the “family” option, the parental variants were used to further reduce the number of variants, but with the additional assumption that the putative variant has to be heterozygous in the child. At this point, 99.99 % of the variants were excluded and only 5 variants remained. The original de novo mutation on chromosome 17 (chr17:72341086G > A) was one of these 5. In the original paper, the number of variants was further reduced by filtering against a second control population and a Sanger sequencing step. An overview of the analysis is provided in Fig. 1. Two remarks have to be made when the “family” option is being used. First of all, each family should be analyzed separately. In addition, unrelated controls should not be included with the “family” option specified as the function would consider them to be parents. This would result in additional variants being filtered, based on assumptions that might not be valid.

### Example 2: coat color in the Labrador Retriever

**Fig. 2:**
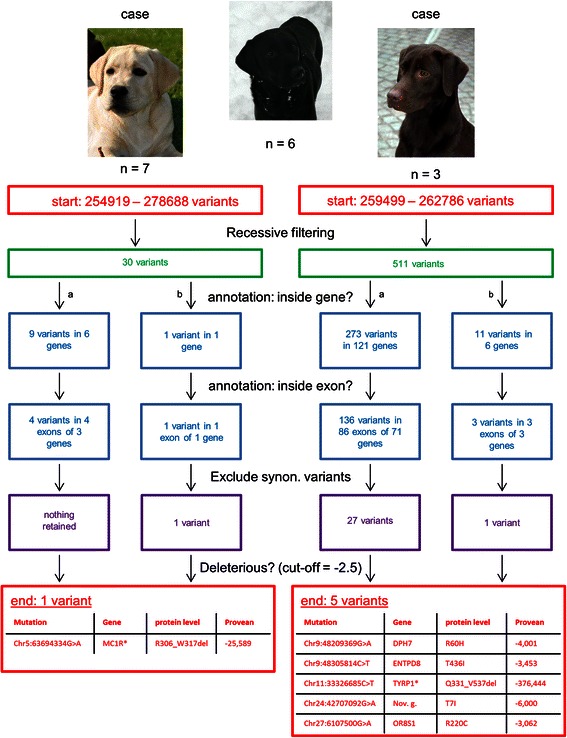
Sequence of heuristic filters to identify causal mutations for coat colors in the Labrador Retriever. The two analysis (yellow (*n =* 7) versus black (*n =* 6) and brown (*n =* 3) versus black (*n =* 6)) were performed separately. The annotation steps were split for the Ensembl Genes (a) and the RefSeq genes (b). The potential effect on the protein was predicted with Provean. The default threshold of −2.5 was used as the cut-off value. * = the causal mutations for brown and yellow coat colors, synon. = synonymous, Nov. g. = novel gene (ENSCAFG00000030103)

In contrast with human studies, WES is not frequently used in domestic species. One of the reasons is likely the limited availability of WES capturing designs. For the dog, the first report on a WES design was published in 2014. The development of new WES designs, are likely to boost disease-association studies in these species [5]. To demonstrate the power of WES studies combined with Mendelian, we revalidated the mutations responsible for the black, brown and yellow coat color in the Labrador Retriever [7–9]. For this analysis, variant data of 16 dogs that were sequenced to validate the exome-plus design, were used [5] . The analysis is detailed in Fig. 2. Based on previous reports and the available pedigree data [see Additional file 2] of the sequenced dogs, it is known that both brown and yellow are inherited recessively as opposed to black [7–9]. For both yellow to black and brown to black, two separate analyses were conducted in parallel. The first step was simple recessive filtering, assuming 100 % detectance and 100 % penetrance. The analysis was continued by two filtering steps based on annotation: at first, only variants that were inside a gene were retained, followed by a second filtering to retain only those variants within known exons. In the final step, only non-synonymous variants were retained. At this point, only one putative variant remained in the comparison of yellow versus black dogs. For the brown versus black analysis, 27 unique putative variants remained and one of them fell within the exon boundaries of both the Ensembl Genes and the RefSeq genes annotation. Further checking learned that both annotations actually referred to the same gene and that the effect on the protein sequence was identical. The two annotations for that specific variant were thus treated as one. To further prioritize the putative variants, the analysis was followed by an assessment of the potential effect of the variant at the protein level with Provean [10]. Finally, the variant responsible for the yellow coat color was identified to be a highly deleterious (Provean score of −25.589) mutation (chr5: 63694334 G > A) introducing a premature stop codon (R306_W317del in *MC1R*). For the brown coat color, the variant which corresponds with the known mutation, was predicted to be the most deleterious (Provean score of −376.444). This mutation (chr11: 33326685 C > T) also results in the introduction of a premature stop codon and removes more than 200 amino-acids from the protein (Q331_V537del in *TYRP1*). None of the other mutations associated with yellow and brown color in the *MC1R* and *TYRP1* genes were present in any of the dogs [11].

Even with a limited number of dogs, it was possible to identify the mutations responsible for the yellow coat color and almost to identify the causal mutation for brown coat color. Importantly, this analysis does not demonstrate the full power of WES for several reasons. First of all, this analysis was conducted without prior filtering to a variant database. For rare disease phenotypes, it is relatively safe to assume that the putative variant has a low MAF in such a database. For a common phenotype such as coat colors, this assumption is not valid and determining an appropriate MAF cut-off will be difficult. In addition, the sequenced dogs were selected to study orthopedic disorders, not coat color. Therefore, the case/control selection was not optimized for our analysis. For example, it is much more interesting to include two full siblings with opposite phenotypes than two siblings with the same phenotype (additional variation reduction of 27.6 % (B. J.G. Broeckx, F. Coopman, G. E.C. Verhoeven, S. De Keulenaer, E. De Meester, V. Bavegems, P. Smets, B. Van Ryssen, F. Van Nieuwerburgh, D. Deforce (in press). Towards the most ideal case-control design with related and unrelated dogs in whole exome sequencing studies. Animal Genetics). Finally, the yellow versus black analysis was somewhat overpowered. A simulation where we gradually included dogs, showed that with 5 yellow dogs and 4 black dogs, we still would have retained the same unique variant [see Additional file 2].

As the attention shifts towards complex disorders, the question is whether Mendelian can be used for those disorders as well. Complex disorders are in essence no more than a combination of genetic and environmental factors that lead to a reduced penetrance and detectance. As Mendelian allows both reduced penetrance and/or detectance, it should be possible technically. However lowering the thresholds will also result in less variants being filtered. Overall, the power of Mendelian for complex disorders will probably be lower compared to simple disorders.

### Comparison with existing software

A limited number of different software packages that filter heuristically are available. Examples are VCFtools [12] and GEMINI [13]. Inside R Bioconductor, the packages VariantFiltering and VariantTools can be used. Compared with these tools, Mendelian has several advantages. GEMINI and VariantFiltering were developed specifically for humans only, which is a disadvantage since WES becomes increasingly popular in a variety of non-model species [2–5]. VariantFiltering does not support multi-allelic variants (variants with more than one alternate allele). Simple analysis tools such as VCFtools and VariantTools only allow for basic analysis (e.g. intersections or complements) and do not support various modes of inheritance [12]. Mendelian is the only package that allows the analysis of variants under reduced penetrance and detectance. To give an idea on the time required when analyzing variant data with Mendelian, some simulations on a standard desktop were added [Additional file 3].

## Conclusions

The identification of one or several causal variant(s) from the vast amount of variant data generated in sequencing experiments, is often based on the sequential use of various filter steps. This software package was designed to provide a species-independent, fast and versatile analysis tool, capable of tackling a wide variety of inheritance models and complicating factors such as genetic heterogeneity and reduced penetrance. We demonstrated its possibilities by reanalyzing a dataset on human intellectual disability and were the first to use WES for the coat color phenotype in the Labrador Retriever without prior mapping. Overall, this package is a valuable tool for causal variant identification in sequencing studies, especially in non-human species were the alternatives are very limited.

## Availability and requirements

**Project name:** Mendelian

**Project home page:** https://github.com/BartBroeckx/Mendelian

**Operating system(s):** Platform independent

**Programming language:** R

**Other requirements:** R version 3.1.0 or higher

**License:** GPL-2

**Any restrictions to use by non-academics:** none

## Additional files

Additional file 1:
**Command line used for example 1 and 2.** The commands used in example 1 and example 2 are depicted in this additional file, together with a brief explanation. (PDF 178 kb)
Additional file 2:
**Pedigree data of the dogs used in the coat color analysis.** In this figure, the familial relation between the dogs used in the analysis, is shown. The color of the squares and circles corresponds with the coat color of the dog (yellow, brown or black). If the coat color is not known, an empty black circle or square was used. □ = male, ○ = female, ^#^ the dogs used in the general analyses, * the 5 yellow dogs and 4 black dogs needed to retain only one variant. (TIFF 81 kb)
Additional file 3:
**Time duration required for processing a variable number of cases and controls with the dominant (Dom) and recessive (Rec) filter (at the nucleotide level) used in example 1 and 2.** Even though each dog had well over 250000 variants, the analysis only took at most around 30 s on a standard desktop (Intel(R) Core(TM) i3-2100 CPU @ 3.10GHz, 4,00 GB RAM, 32-bit Windows 7). The inclusion of controls decreases the computing time through a reduction of the number of variants in the cases. The recessive filter outperforms the dominant filter here as the size of the data frames is reduced by the exclusion of heterozygous variants. (TIFF 108 kb)

## Abbreviations

MAF: Minor allele frequency
VCF: Variant call format
WES: Whole exome sequencing
