# Supplementary material for: An heuristic filtering tool to identify phenotype-associated genetic variants applied to human intellectual disability and canine coat colors
Source: BMC Bioinformatics. 2015 Nov 19;16:391. doi: 10.1186/s12859-015-0822-7 (PMC4656174; doi:10.1186/s12859-015-0822-7)
Supplement: Additional file 1: — Command line used for example 1 and 2. The commands used in example 1 and example 2 are depicted in this additional file, together with a brief explanation. (PDF 178 kb) [file 12859_2015_822_MOESM1_ESM.pdf]

Additional file 1: command line used for example 1 and 2:

Installation of "Mendelian" in R:

```
devtools::install_github("BartBroeckx/Mendelian", build_vignettes=TRUE)
```

By setting build_vignettes to TRUE, the vignette (= manual) is downloaded as well. The vignette can be accessed by typing:

```
vignette("Mendelian-vignette")
```

Example 1:

1. Input: example of reading in the .vcf files in R:

```
patient1<-read.table("a")

parent1 <- read.table("b")

parent2 <- read.table("c")
```

2. Preparing the .vcf files for further processing with a filtering to retain only those variants that passed the quality filters of the GATK pipeline:

```
patient1proc<-VCFfile(patient1, "V10", filter=TRUE, "PASS")

parent1proc <- VCFfile(parent1, "V10", filter=TRUE, "PASS")

parent2proc <- VCFfile(parent2, "V10", filter=TRUE, "PASS")
```

3. Reading in the dbSNP data:

```
dbSNP <- read.table("dbSNP135", header=TRUE, sep="\t")
```

4. Preparing the dbSNP for filtering:
    a. Deciding how many processors to use to prepare the dbSNP:

```
library(doParallel)

registerDoParallel()

nproc <-getDoParWorkers()
```

b. The actual filtering against a variant database with the MAF unspecified:

dbSNPfilter <- prepvarpar(dbSNP,,”refNCBI”, nproc)

Remark: an unspecified MAF gives the same result as setting MAF = 0, e.g.:

dbSNPfilter <- prepvarpar(dbSNP,0,”refNCBI”, nproc)

5. Removing all the variants present in the dbSNP from the variants in the patient:

filtered <- varfilter(patient1proc,dbSNPfilter)

6. Trio filtering:

nDom("filtered", c("parent1proc", "parent2proc"), "Ps-F")

<u>Example 2:</u>

1. Input: example of reading in of one .txt output file from CLC Genomcics Workbench in R:

a<-read.table("a", header=TRUE, sep="\t")

2. Standard recessive filtering (with three cases and three controls):

b <-nRec(c("a", "b", "c”), c("d", "e", "f"))

3. Annotation:
    a. reading in the RefSeq Genes annotation as downloaded from the UCSC table browser:

RefBED<-read.table("bed", sep="\t", header=FALSE)

    b. the actual annotation process with removal of all variants that do not fall inside the RefSeq regions

out<- annot(a,RefBED, type="BED", nomatch=FALSE, CLC=TRUE)

Additional examples for each function are provided together with the installation of the R-package. They can be accessed by combining “?” with the function you require information for, e.g. “?annot”
