# Supplementary figures and images for: An heuristic filtering tool to identify phenotype-associated genetic variants applied to human intellectual disability and canine coat colors
Source: BMC Bioinformatics. 2015 Nov 19;16:391. doi: 10.1186/s12859-015-0822-7 (PMC4656174; doi:10.1186/s12859-015-0822-7)

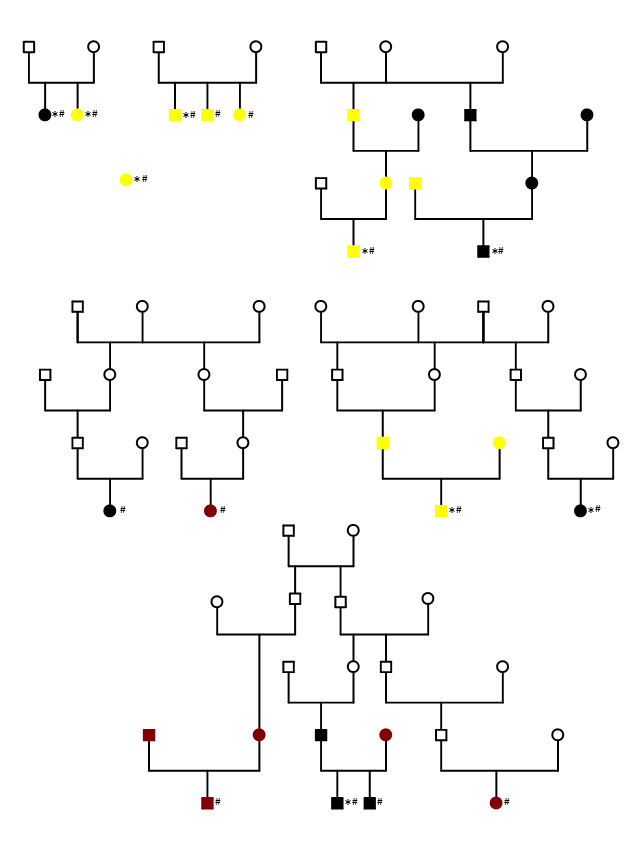

Supplement: Additional file 2: — Pedigree data of the dogs used in the coat color analysis. In this figure, the familial relation between the dogs used in the analysis, is shown. The color of the squares and circles corresponds with the coat color of the dog (yellow, brown or black). If the coat color is not known, an empty black circle or square was used. □ = male, ○ = female, # the dogs used in the general analyses, * the 5 yellow dogs and 4 black dogs needed to retain only one variant. (TIFF 81 kb) [file 12859_2015_822_MOESM2_ESM.tiff]

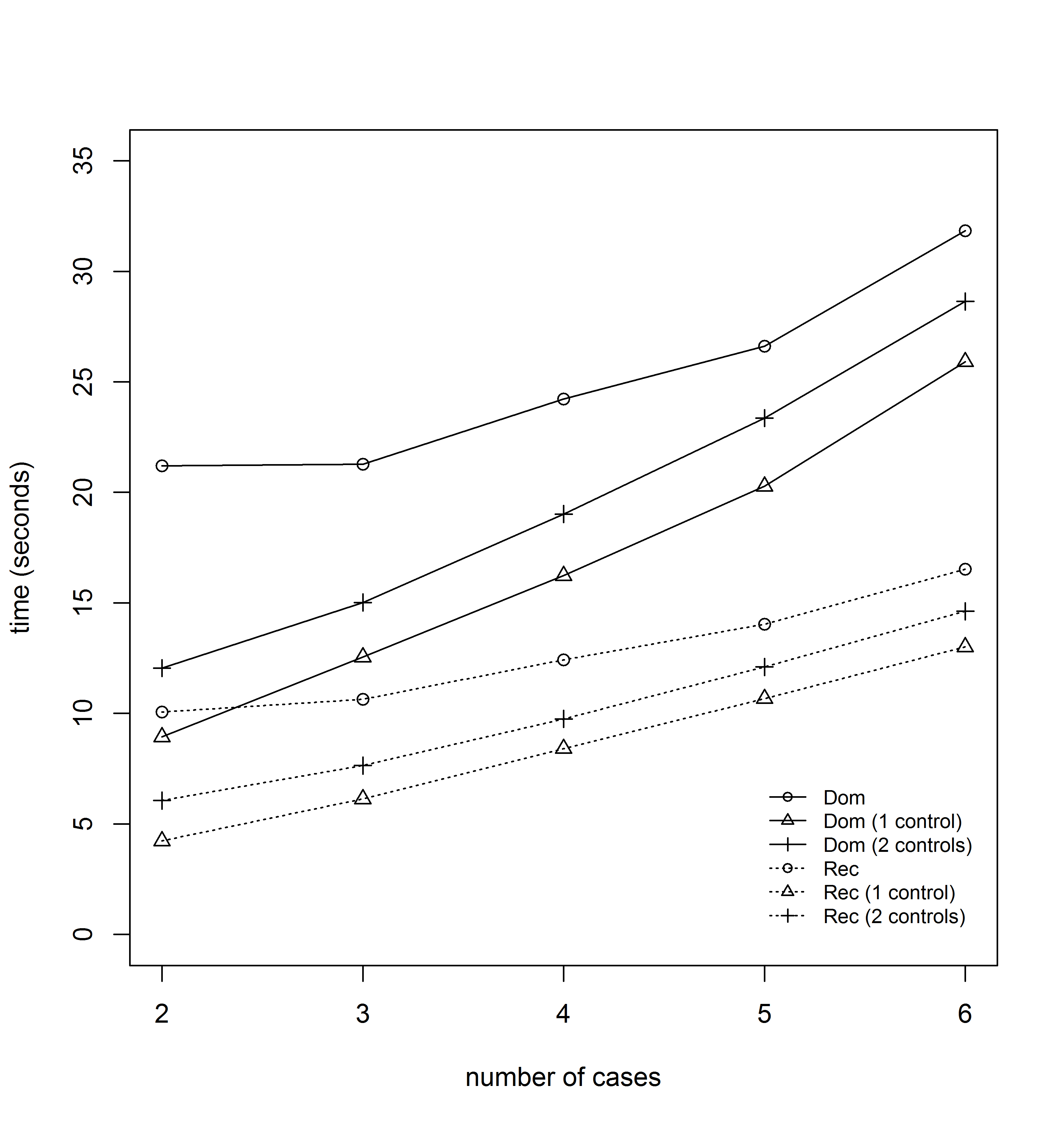

Supplement: Additional file 3: — Time duration required for processing a variable number of cases and controls with the dominant (Dom) and recessive (Rec) filter (at the nucleotide level) used in example 1 and 2. Even though each dog had well over 250000 variants, the analysis only took at most around 30 s on a standard desktop (Intel(R) Core(TM) i3-2100 CPU @ 3.10GHz, 4,00 GB RAM, 32-bit Windows 7). The inclusion of controls decreases the computing time through a reduction of the number of variants in the cases. The recessive filter outperforms the dominant filter here as the size of the data frames is reduced by the exclusion of heterozygous variants. (TIFF 108 kb) [file 12859_2015_822_MOESM3_ESM.tiff]
